# Bone pain in fibrous dysplasia does not rely on aberrant sensory nerve sprouting or neuroma formation

**DOI:** 10.1093/jbmr/zjaf066

**Published:** 2025-05-05

**Authors:** Biagio Palmisano, Chiara Tavanti, Giorgia Farinacci, Giorgio Gosti, Marco Leonetti, Samantha Donsante, Giuseppe Giannicola, Natasha Appelman-Dijkstra, Alessandro Corsi, Ernesto Ippolito, Mara Riminucci

**Affiliations:** Department of Molecular Medicine, Sapienza University of Rome, 00161 Rome, Italy; Department of Molecular Medicine, Sapienza University of Rome, 00161 Rome, Italy; Department of Molecular Medicine, Sapienza University of Rome, 00161 Rome, Italy; Soft and Living Matter Laboratory, Institute of Nanotechnology, Consiglio Nazionale delle Ricerche, 00185 Rome, Italy; Center for Life Nano- and Neuro-Science, Italian Institute of Technology, 00161 Rome, Italy; Soft and Living Matter Laboratory, Institute of Nanotechnology, Consiglio Nazionale delle Ricerche, 00185 Rome, Italy; Center for Life Nano- and Neuro-Science, Italian Institute of Technology, 00161 Rome, Italy; Department of Molecular Medicine, Sapienza University of Rome, 00161 Rome, Italy; Tettamanti Center, Fondazione IRCCS San Gerardo dei Tintori, 20900 Monza, Italy; Department of Anatomical, Histological, Medico Legal and Orthopaedic Sciences, Sapienza University of Rome, 00161 Rome, Italy; Division of Endocrinology, Department of Internal Medicine, Leiden University Medical Centre, 2333 Leiden, The Netherlands; Department of Molecular Medicine, Sapienza University of Rome, 00161 Rome, Italy; Department of Orthopaedic Surgery, University of Rome Tor Vergata, 00133 Rome, Italy; Department of Molecular Medicine, Sapienza University of Rome, 00161 Rome, Italy

**Keywords:** musculoskeletal pain, mouse models, behavior, bone innervation, fibro-osseous lesions, benign bone tumors, skeletal dysplasia, McCune–Albright syndrome

## Abstract

Bone pain is a major symptom of many skeletal disorders. Fibrous dysplasia (FD) is a genetic disease with mono or polyostotic skeletal phenotype due to the post-zygotic occurrence of the causative Gsα mutation. Bone pain in FD often associates with skeletal deformities and fractures or nerve impingement by the pathological tissue. However, even in the absence of complications, FD patients often complain of a chronic pain that does not correlate with their disease burden. Multiple hypotheses have been made to explain this pain. However, its pathogenetic mechanisms remain, as yet, largely unexplored. In this study, we first demonstrate that the FD mouse model *EF1α-Gsα^R201C^* develops behavioral impairments and altered response to nociceptive stimuli that, as in FD patients, do not correlate with their skeletal disease burden, thus providing a reliable model to study bone pain in FD. Then, we show that in *EF1α-Gsα^R201C^* mice, the overall pattern of skeletal innervation is preserved and that within FD lesions, sensory fibers are variably and focally distributed, mainly at perivascular sites. Finally, we provide the first analysis of a series of human FD bone biopsies showing that, within the lesional tissue, sensory nerve fibers are few despite the rich vascular network and appear to be well-organized. These data show that, albeit sensory nerve fibers are found within FD lesions, bone pain in humans and functional impairment in mice are not associated to pathological sensory nerve sprouting or formation of neuromas in the Gsα-mutated skeleton.

## Introduction

Bone pain is a common and severe symptom of many bone diseases, from genetic disorders to cancer metastasis. As in other organs, pain sensation in bone mainly results from signals collected by the peripheral nervous system upon stimulation (nociceptive pain) or damage (neuropathic pain) of different types of nerves and are then processed by the central nervous system (CNS). Studies in humans^1^ and animal models^2^^,^^3^ showed that bone pain is conducted mostly by A-delta and C nerve fibers. The former are myelinated and express the nerve growth factor (NGF), receptor tropomyosin kinase A (TRKA) and the 200 kDa neurofilament (NF200); the latter are unmyelinated and express TRKA and calcitonin gene-related peptide (CGRP).^4^ These fibers are found with decreasing density in the periosteum, bone marrow, and cortical bone. In the periosteum they can detect mechanical stimuli, for example, in bone fractures, while in the bone marrow they conduct sensory information due to compression, for example, by cancer cells. In some pathological conditions, bone nerve fibers may undergo sprouting processes or form neuroma-like structures that further increase the sensation of pain.^5^

Bone pain is a major clinical aspect of fibrous dysplasia (FD), a rare, skeletal disease caused by gain-of-function mutations of the Gsα gene, most commonly at the codon R201 (R201C, R201H).^6^ Due to the post-zygotic occurrence of the mutations, FD patients are clinically heterogeneous with different mono or polyostotic patterns of skeletal involvement and, in the most severe cases, different types of extra-skeletal manifestations (McCune–Albright syndrome, MAS).^7^ The development of FD lesions occurs through the replacement of normal bone and marrow by fibrous tissue containing tiny and hypo-mineralized bone trabeculae. A previous report showed that both adults and children with FD/MAS may experience pain^8^ and an analysis performed on a large cohort of adult FD/MAS patients, demonstrated the presence of both nociceptive and neuropathic pain.^9^ Furthermore, a recent study reported that pain in FD may also show neurobiological and neuropsychological features.^10^ Despite the presence of pain and its variable and often unsatisfactory response to pharmacological treatments such as anti-resorptive, anti-inflammatory drugs and opiates,^11–14^ the mechanisms of pain sensation in FD patients are, as yet, incompletely understood. Furthermore, although it has been speculated that the pathological fibro-osseous tissue associates with abnormal nerve fiber growth,^11^ no data are currently available on the pattern of innervation of FD lesions.

The *EF1α-Gsα^R201C^* mouse model reproduces the elementary tissue changes of human FD, shows time-dependent heterogeneous patterns of skeletal involvement as FD patients, and has been proved to be a useful tool for investigating the effect of pharmacological strategies on the development and progression of the disease.^15–17^

Having observed that *EF1α-Gsα^R201C^* mice can manifest behavioral signs that in rodent are related to pain, we hypothesize that their well-being could be affected by the development of FD lesions. We also asked whether pain in *EF1α-Gsα^R201C^* mice and FD patients associates with aberrant growth and/or organization of nerve fibers in developing and fully established bone lesions.

Here, we report that *EF1α-Gsα^R201C^* mice develop a behavioral impairment and an altered nociceptive response that, as in humans,^18^ do not correlate with their skeletal disease burden. Moreover, by generating the *BAF53b-GFP;EF1α-Gsα^R201C^* reporter mouse model with pan-neuronal GFP expression along with immunolocalization studies, we show that the sensory innervation pattern of mouse Gsα-mutated skeletal compartments and FD lesions does not show morphological abnormalities. Finally, we report for the first time that human FD lesions are poorly innervated, sensory nerve fibers are few and without evidence of abnormal organization, thus providing an important insight into the pathophysiology of pain in FD/MAS.

## Materials and methods

### Generation of experimental mice

*The EF1α-Gsα^R201C^* mouse model was generated previously.^19^  *Tg(Actl6b-Cre)4092Jiwu/J (BAF53b-Cre)* mice, expressing *Cre* recombinase under the control of the pan-neuronal *Actl6b* promoter (*BAF53b-Cre*),^20^ and the reporter line *B6.129(Cg)-Gt(ROSA)26Sortm4(ACTB-tdTomato,-EGFP)Luo/J* (*R26-mTmG)*^21^ were purchased from The Jackson Laboratory. *BAF53b-Cre *mice** were crossed with *R26-mTmG* and with *EF1α-Gsα^R201C^* mice to generate *BAF53b-GFP;EF1α-Gsα^R201C^* mice, a triple transgenic model of FD expressing GFP in all nerve fibers. This strain was maintained at a mixed background (B6;FVB), therefore only littermates were used as controls. Genotyping was performed by PCR using MyTaq DNA Polymerase (Meridian Bioscience Inc.) using specific oligonucleotide primers (Table S1).

Mice were maintained in cabin-type isolators at standard environmental conditions (temperature 22 °C-25 °C, humidity 40%-70%) with 12:12 dark/light photoperiod. Food and water were provided ad libitum.

All studies were performed in compliance with relevant Italian laws and Institutional guidelines and all procedures were IACUC approved.

### Behavioral tests

Behavioral analyses were carried out during the dark phase, starting from 6 pm, to avoid any potential behavioral inhibition of the resting light phase. We examined male and female mice, and findings are reported for both sexes. Experimental groups were generated by random selection of transgenic mice, to allow the inclusion of *EF1α-Gsα^R201C^* mice with diverse skeletal phenotypes and avoid potential biases. The number of mice used for each test are reported in the figure legends. All procedures were performed by a single investigator, who was blinded during animal allocation and handling, and endpoint behavioral measurements.

#### Burrowing test

Burrowing is an adaptive behavior conserved across many rodent species and is an expression of the animal well-being. The burrowing test is considered a sensitive test simulating the “activities of daily living” in humans, which are often impeded by pain states. This test assays the spontaneous ability of mice to empty a tube filled with food pellets.^22^

The test was performed as previously described^23^ with minor modifications. To generate the burrow tubes, a PVC downpipe with 68 mm diameter (OBI Italia) was cut into 20-cm-long lengths. One end of each tube was sealed with an aluminum lid and the other end was elevated about 3 cm off the floor with 5-cm-long machine screws. Habituation was carried out overnight (ON) and was conducted in group by placing 4-5 mice into a 26 (length) × 23 (width) × 17 (height) cm cage containing the burrow tube filled with food pellets. Forty-eight hours later each mouse was transferred into a cage containing the tube filled with 300 g of pellets. The evaluation of the burrowing activity was performed by weighing the food remaining in the tube at different time points. The first evaluation was performed after 2 h (2H), then the tube was filled again with pellets and a second measurement was taken the next morning (ON).

#### Nesting test

Nest building is a behavior that mice instinctively perform for housing their pups, thermoregulation and comfort. It relies on the functional integrity of mouse sensory and motor systems. Therefore, the nesting test is used to assess the general well-being of mice.^22^

Mice were transferred into individual cages containing 4.5 g of orderly placed shredded paper as nesting material without any other environmental enrichment. The following morning, the nest was photographed and given a score from 1 to 5^24^ based on the organization and the amount of paper used to build it. A detailed table is reported in Figure S1A.

As nest construction reflects a complex interaction between the animal and its environment, in some cases the results did not fit perfectly one of the scores. In these cases, a half point was either subtracted or added to the score.^24^ For example, some mice built a score 5 nest but used a bit less than 90% of the paper thus receiving a score of 4.5.

#### Open field test

This test allows to evaluate the exploratory behavior and general locomotor activity in rodents.^25^ Nine-month-old male mice were transferred to the testing room at short distance from the holding facility. Each mouse was placed into the center of a 26 (length) × 15.5 (width) × 14 (height) cm cage and allowed to explore it freely for 5 min for habituation. Then, it was monitored for 2 min by video tracking. Videos were filmed using a high-end single-cam device, featuring a 12Mp 1/2.55″ sensor (mounted on an Apple iPh1 XR), at 1920 × 1080 resolution, 30 frames/s and analyzed by Python code developed in house using Numpy, Matplotlib, Pandas, and OpenCV. The mouse body was segmented using the HSV color space. The code is available online at https://github.com/ggosti/HueMouse. The overall motor activity was quantified as the total traveled distance.

#### Tail flick test

This test is one of the most used and oldest nociceptive tests.^22^ It was described originally as a measure of pain threshold in rodents.^26^ It measures the latency, in seconds, for tail flick reflex following tail exposure to a radiant heat stimulus.

Each mouse was gently held and positioned on the tail flick apparatus (Ugo Basile) with the tail placed over a small window emitting a beam of infrared energy at 65% intensity. The heat stimulus was applied on the mid portion and on the tip of the tail and the latency of the tail flicking was automatically scored by an optic fiber.

### X-ray analyses and disease burden score

The disease burden score was assessed on radiographic images by an arbitrary scoring system. Images were taken by Faxitron MX-20 Specimen Radiography System (Faxitron X-ray Corp.) set at 24-25 kV for 6-8 s under anesthesia with a mixture of Zoletil 50/50 (Virbac SA) and Rompun (Bayer). A value from 0 to 3 was assigned to each bone segment based on the area occupied by FD lesions as reported in Figure S2A. Specifically, score 0 was assigned when no FD lesions were detected; score 1 when lesions involved less than 25% of the bone segment; score 2 when FD lesions occupied 25%-50% and score 3 when more than 50% of the bone segment was affected. The sum of the individual skeletal segment scores was considered as the final score for each mouse.

### Histology and immunofluorescence of mouse samples

Mice were euthanized by carbon dioxide inhalation and the skeletal segments were fixed with 4% formaldehyde for 6 h at 4 °C. After decalcification in 0.5 M EDTA for 48-96 h, the samples were embedded in porcine gelatin as described previously^27^ and 60-μm-thick sections were cut. To visualize the endogenous fluorescence, sections were thawed at room temperature (RT), rehydrated with phosphate-buffered saline (PBS) and stained for 30 min with the nucleic acid stain TO-PRO3 (T3605, Thermo Fisher Scientific) for visualization of nuclei.

For immunofluorescence, sections were rehydrated in PBS with 0.3% Triton for 10 min and incubated with 5% goat serum for 30 min at RT. Anti-NF200 antibody (N4142, Sigma-Aldrich), anti-TRKA antibody (AF1056, R&D System), and anti-CGRP antibody (ab81887, Abcam) were applied at a dilution of 1:500, 1:50, and 1:1000 in PBS, respectively. Anti-SCA-1 and anti-CD31 antibodies (122501 and 102501, Biolegend) were applied at a dilution of 1:100. Incubation of primary antibodies was carried out ON at 4 °C. Sections were then washed with PBS, incubated at RT for 1.5 h with appropriate secondary antibodies (A-11034 and A-1178, Thermo Fisher Scientific), and stained with TO-PRO-3 to visualize nuclei. Images were acquired with Leica Confocal Microscope (Leica Biosystems). Z-stacked images were analyzed with Fiji ImageJ software.^28^

The analysis of nerves and blood vessels was performed on randomly selected areas in caudal vertebrae from *BAF53b-GFP* and *BAF53b-GFP;EF1α-Gsα^R201C^* mice. Nerve fibers and blood vessels were manually traced to assess the ratio between their respective lengths.

### Histology and immunohistochemistry of human samples

FD and control samples were used following patients’ written informed consent and with the approval of the local Institutional Review Board.

Histological studies included 13 FD archival paraffin embedded bone biopsies used in our previous study.^29^ Samples were collected from femurs and tibiae of 7 FD/MAS patients, whose clinical data are reported in Table 1. Patients complained of pain at affected sites at the time of surgery, although no assessment through standard evaluation scales was performed. One patient (biopsies 9 and 10) was treated with bisphosphonates, 4 did not receive any anti-resorptive therapy while for 2 patients, information about treatments was not available.

**Table 1:** Data on the FD patients and samples included in the histological study.

**ID**	**Patient**	**Gender and age**	**Mutation**	**Clinical diagnosis**	**Reported pain**	**Receiving BPs**	**Site of bone biopsy or surgery**	**Area of the biopsy (mm** ^**2**^ **)**
**Biopsy 1**	FD 1	M/20 yr	R201H	MAS	Yes	No	Femur	51.99
**Biopsy 2**		M/23 yr				No	Tibia	291.12
**Biopsy 3**	FD 2	F/18 yr	R201C	MAS	Yes	/	Femur	43.46
**Biopsy 4**	FD 3	F/19 yr	R201C	MAS	Yes	/	Femur	36.03
**Biopsy 5**	FD 4	M/14 yr	R201H	Polyostotic FD	Yes	No	Femur	138.63
**Biopsy 6**							Femur	87.26
**Biopsy 7**	FD 5	M/11 yr	R201H	MAS	Yes	No	Femur	46.34
**Biopsy 8**							Femur	9.73
**Biopsy 9**	FD 6	F/16 yr	R201C	MAS	Yes	Yes	Femur	47.32
**Biopsy 10**							Femur	132.88
**Biopsy 11**	FD 7	M/7 yr	R201H		Yes		Femur	164.99
**Biopsy 12**				Polyostotic FD		No	Femur	101.62
**Biopsy 13**							Femur	260.25

Abbreviation: BPs, bisphosphonates. Information about treatment was not available for patients FD2 and FD3.

Control samples were harvested from the tibia of a 19-yr-old male healthy donor (HD) and from the unaffected fibula of the FD 1 patient during corrective surgery.

After fixation with 4% formaldehyde and decalcification in 0.5 M EDTA, biopsies were embedded in paraffin according to standard procedure. One sample was embedded in porcine gelatin. Histology was performed on 3-μm-thick paraffin sections after H&E and Sirius red (SR) staining. Immunohistochemistry was performed on paraffin sections after heat-induced epitope retrieval (98 °C for 30 min) in citrate buffer for NF200 and CGRP, and Tris-EDTA buffer for PGP9.5. Sections were exposed to 10% bovine serum albumin (BSA) in PBS for 30 min at RT to block non-specific binding.

PGP9.5 immunolocalization was performed using a rabbit anti-human antibody (SAB4503057, Sigma-Aldrich), applied at the dilution of 1:100 for 1 h at 37 °C. Rabbit anti-human TH antibody (NB300-109, Novus Biologicals) was applied at the dilution of 1:50 for 2 h at RT. Sections were then incubated for 30 min with a biotin-conjugated swine anti-rabbit antibody (E0353, Agilent Dako), 1:500 in 1% BSA-PBS, then exposed to 0.3% H_2_O_2_ in methanol for 10 min at RT for endogenous peroxide enzyme blocking and finally incubated for 30 min in horseradish peroxidase (HRP)-conjugated streptavidin (P0397, Agilent Dako) 1:1000 in PBS.

CGRP immunolocalization was carried out using a mouse anti-human antibody (ab81887, Abcam), applied at the dilution of 1:200 for 2 h at RT. After incubation with the primary antibody, sections were exposed for 30 min to biotin-conjugated rabbit anti-mouse antibody (E0464, Agilent Dako) 1:300 in PBS, to 0.3% H_2_O_2_ in methanol for 10 min at RT and then to HRP-streptavidin for 30 min as described above. The HRP reaction was developed using 3,3′-diaminobenzidine tetrahydrochloride kit (SK-4105, Vector Laboratories).

For NF200 and S100 staining, sections were incubated with specific antibodies (NCL-L-NF200-N52 and NCL-L-S100-167, respectively, Leica Biosystems) 1:100 for 30 min at 25 °C in an automated Leica BOND III system (Leica Biosystems). Staining was developed using BOND Polymer Refine Detection (DS9800, Leica Biosystems).

NF200 immunofluorescence was performed on 60-μm-thick gelatin sections using the anti-NF200 antibody as described for mouse sections.

To estimate the number of PGP9.5 and NF200-positive nerves in FD bone biopsies, we performed a count that was normalized on the whole biopsy area (Table 1) and expressed as nerve density (1/mm^2^). A nerve in the form of a cluster of axons was counted as one single entity.

### Gene expression analysis

Samples from the FD patients and HDs, the latter collected as surgical waste during orthopedic elbow surgery, were frozen and stored for gene expression analysis. Total RNA was isolated using the TRI Reagent (Thermo Fisher Scientific) and reverse transcribed using PrimeScript RT Reagent Kit (Takara Bio). Quantitative PCR (qPCR) was performed on a 7500 Fast Real-Time PCR System (Thermo Fisher Scientific) using PowerUP Sybr Green (Thermo Fisher Scientific) and specific primers (Table S1). RNA 18s ribosomal N5 (*RNA18SN5*) expression was used for normalization.

### Statistical analysis

An a priori analysis was conducted using G^*^Power software (version 3.1.9.6, HHU) for sample size estimation, based on data obtained from a pilot burrowing test study, in which we compared WT (*n* = 9) to EF1α-Gsα^R201C^ (*n* = 9) female mice of 9 mo of age. The effect size in the pilot study was 0.32. With a power set at 80% and α error probability of 0.05, the minimum sample size needed in each sex with this effect size was *n* = 105 for an ANOVA statistical test (number of groups considered: 8, ie, 2 genotypes and 4 different age groups).

Non-parametric Mann–Whitney test was used to compare 2 groups when the population of data did not have a normal distribution while unpaired Student’s *t*-test was used when the 2 populations were normally distributed. Two-way ANOVA test was used to detect statistical differences in the behavioral tests between WT and *EF1α-Gsα^R201C^* mice at different ages. Correlation analyses were conducted using the Pearson test and coefficient r and *p*-value were showed. In all experiments a *p*-value <.05 was considered statistically significant. Graphs and statistical analyses were performed using GraphPad Prism 10 (GraphPad Software).

## Results

### EF1α-Gsα^R201C^ mice displayed altered behavior and impaired well-being

**Figure 1:**
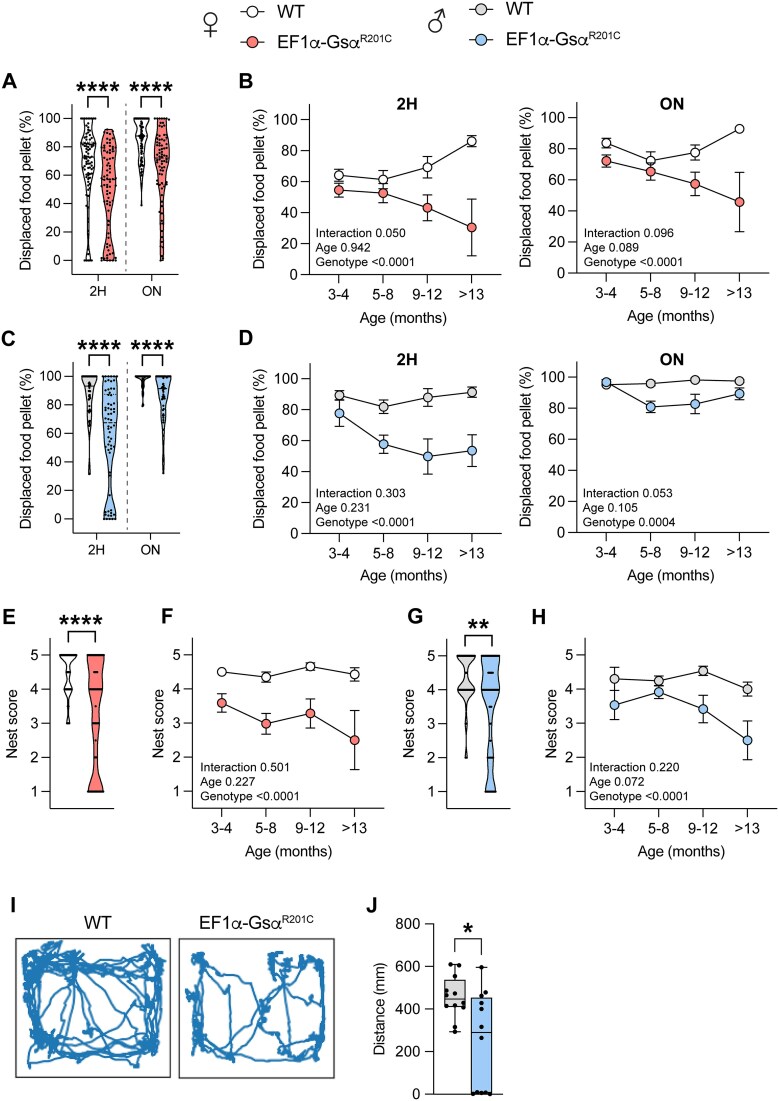
Assessment of spontaneous pain-like behavior. (A) Burrowing test results obtained from 2 h (2H) and overnight (ON) experiments on female mice. WT *n* = 90; *EF1α-Gsα^R201C^ n* = 78, Mann–Whitney test ^****^p < .0001. (B) Age stratification of burrowing test results from female mice. *p*-values from the two-way ANOVA analysis are reported in each graph. (C) Burrowing test results obtained from 2H and ON experiments on male mice. WT *n* = 56; *EF1α-Gsα^R201C^ n* = 62, Mann–Whitney test ^****^p < .0001. (D) Age stratification of burrowing test results from male mice. *p*-values from the two-way ANOVA analysis are reported in each graph. (E) Nesting test results from experiments performed on female mice. WT *n* = 83; *EF1α-Gsα^R201C^ n* = 77, Mann–Whitney test ^****^p < .0001. (F) Age stratification of nesting test results from female mice. *p*-values from the two-way ANOVA analysis are reported in each graph. (G) Nesting test results from experiments performed on male mice. WT *n* = 67; *EF1α-Gsα^R201C^ n* = 70, Mann–Whitney test ^****^p < .0001. (H) Age stratification of nesting test results from male mice. *p*-values from the two-way ANOVA analysis are reported in each graph. (I) Representative video tracking images of open field test performed on 9-mo-old male mice. (J) Total distances covered by the mice during the 2-min open field test. WT *n* = 12; *EF1α-Gsα^R201C^ n* = 12, Mann–Whitney test ^*^p < .05.

Evaluation of *EF1α-Gsα^R201C^* mouse well-being was carried out using standard behavioral tests. Burrowing capacity was significantly reduced in *EF1α-Gsα^R201C^* compared to WT mice, in both males and females (Figure 1A-D). Age stratification of mice revealed that burrowing behavior in *EF1α-Gsα^R201C^* female mice was reduced as early as 3 mo of age (Figure 1B), whereas differences in males were observed starting at 5 mo of age (Figure 1D). Two-way ANOVA simple main effects analysis indicated that genotype, rather than age, had a statistically significant effect on the burrowing capacity in both female and male mice (Figure 1B and D).

Similarly, the capacity of both female and male *EF1α-Gsα^R201C^* mice to build a well-formed nest was significantly compromised (Figure 1E-H). Nests built by WT mice were three-dimensional, well assembled, and developed in height (Figure S1B). In contrast, most of the nests made by *EF1α-Gsα^R201C^* mice were flat and disorganized and in some of them the paper strings were chewed by the mice (Figure S1B). Stratification of results according to age demonstrated lower nesting score in *EF1α-Gsα^R201C^* mice compared to WT at all ages and in both sexes (Figure 1F and H). Two-way ANOVA simple main effects analysis showed that genotype, but not age, had a statistically significant effect on this behavioral test, in both female and male mice (Figure 1F and H). Furthermore, the analysis of mouse ambulation performed by open field test indicated that *EF1α-Gsα^R201C^* mice had an impaired locomotor activity compared to WT (Figure 1I and J). Altogether, these data demonstrated that well-being in *EF1α-Gsα^R201C^* mice was compromised, possibly due to the development of a pain-like behavior.

### EF1α-Gsα^R201C^ mice showed delayed nociceptive response

**Figure 2:**
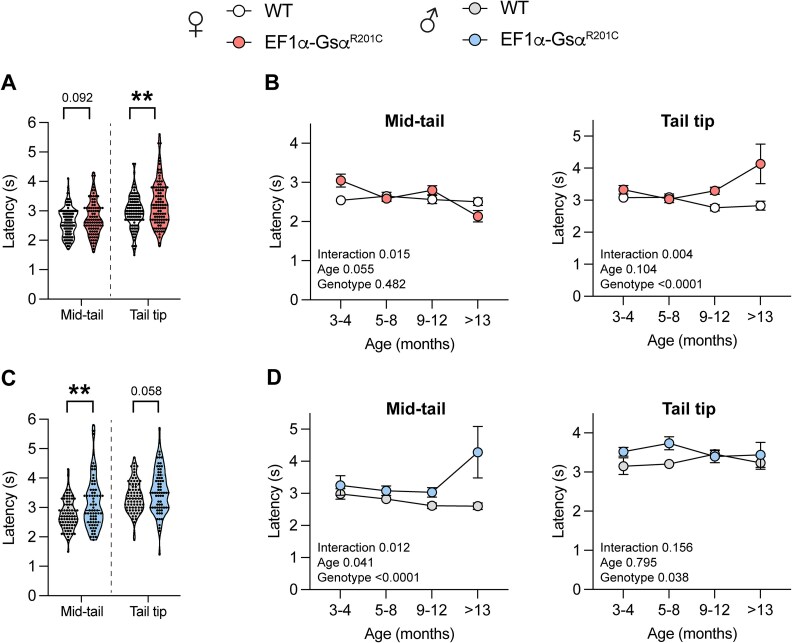
Assessment of nociceptive response. (A) Tail flick test results obtained on the mid-tail and tail tip of female mice. WT *n* = 110; *EF1α-Gsα^R201C^ n* = 99; Welch’s *t* test ^**^p < .01 or exact *p*-value is reported. (B) Age stratification of tail flick test results from female mice. *p*-values from the two-way ANOVA analysis are reported in each graph. (C) Tail flick test results obtained on the mid-tail and tail tip of male mice. WT *n* = 80; *EF1α-Gsα^R201C^ n* = 71; Welch’s *t* test ^**^p < .01 or exact *p*-value is reported. (D) Age stratification of tail flick test results from male mice. *p*-values from the two-way ANOVA analysis are reported in each graph.

In order to assess if FD lesion development in mice altered their nociceptive response, we used the tail flick test. The test was performed on 2 segments of the mouse tail, the mid-tail and the tail tip, and the results were analyzed independently. Interestingly, we observed an overall longer latency of response to the heat stimulus in *EF1α-Gsα^R201C^* compared to WT mice (Figure 2A-D). This result was more consistent in males in which the reaction time was increased compared to WT at both mid-tail and tail tip (Figure 2C), while in females a statistically significant difference was observed only at the tail tip (Figure 2A). Two-way ANOVA analysis revealed that, except for the mid-tail measures in females, genotype had a statistically significant effect on the tail flick test (Figure 1B and D).

These data demonstrated that *EF1α-Gsα^R201C^* mice did not show increase nociception and rather displayed a delayed response to stimulation of sensory fibers.

### Behavioral test scores in EF1α-Gsα^R201C^ mice did not correlated with FD disease burden

To assess the correlation between the skeletal disease burden of *EF1α-Gsα^R201C^* mice and their age, body weight, and pain-like behavior we developed a disease scoring system based on radiographic analysis (Figure S2A). Pearson correlation coefficient revealed a significant, positive correlation between the skeletal burden score and the mouse age (Figure S2B). This confirmed the validity of the disease scoring method as the number and severity of FD lesions in *EF1α-Gsα^R201C^* mice increase with age.^19^ Accordingly, a positive, although not statistically significant, correlation was also observed between skeletal burden and body weight (Figure S2C), as the latter tends to increase with age in rodents. This finding also indicates that albeit *EF1α-Gsα*^*R201C*^ mice are markedly lighter than WT (Figure S2D), they retain the ability to gain weight during aging in spite of the progression of the disease (Figure S2D).

Then we analyzed the correlation between the mouse behavior and the skeletal disease burden scores. Surprisingly, no correlation was found between the results from the burrowing, nesting and open field tests and the extent of skeletal involvement by the disease (Figure S2E-G). Interestingly, we observed that mice showing the same disease burden score could present variable burrowing, nesting or locomotor capacity. For instance, mice with lesions detected only in tail could show greater functional impairment than mice with many affected skeletal segments and high degree of disease burden (Figure S2E and F). As in the other tests, no significant correlation was observed between the skeletal disease burden score and the results of the tail flick test (Figure S2H). Finally, we did not find a correlation among the results of the different behavioral tests, except between the burrowing and open field analyses (Figure S2I), suggesting that the test results did not reflect a general malaise of the mice.

### *BAF53b-GFP;EF1α-Gsα* ^*R201C*^ mice did not show pathological nerve sprouting in FD lesions

**Figure 3:**
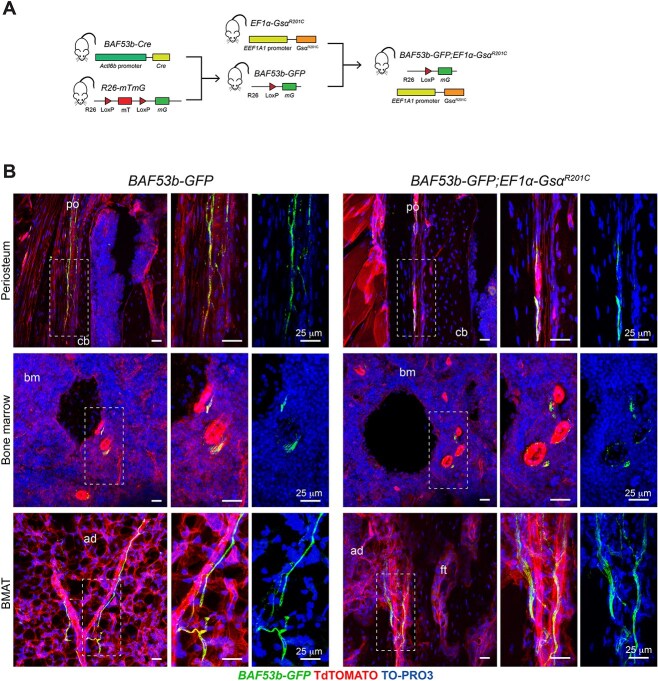
Localization of GFP-positive nerve fibers in the periosteum and bone marrow of 8-mo-old *BAF53b-GFP* (controls) and *BAF53b-GFP;EF1α-Gsα^R201C^* mice. (A) Generation of the triple transgenic *BAF53b-GFP;EF1α-Gsα^R201C^* mouse model of FD. (B) Nerve fibers distribution in the fibrous layer of the periosteum, hematopoietic bone marrow and in the bone marrow adipose tissue (BMAT) of caudal vertebrae. No overall differences between *BAF53b-GFP* and *BAF53b-GFP;EF1α-Gsα^R201C^* mice are observed. Femoral periosteum and BMAT panels are from longitudinal sections, femoral hematopoietic bone marrow images are from transverse sections. Z-stacks of the confocal images are 60 μm thick. Abbreviations: po, periosteum; cb, cortical bone; bm, bone marrow; ad, bone marrow adipocytes; ft, fibrous tissue.

To investigate whether the pattern of innervation of mouse bones was altered in Gsα-mutated mice, either as a consequence of FD lesion development or as a cell autonomous effect of the mutation in the peripheral nervous system, we set up lineage tracing experiments. We generated a triple mutant mouse model, by crossing *EF1α-Gsα^R201C^* mice with *BAF53b-Cre* mice and *R26-mTmG mice*, to obtain *BAF53b-GFP;EF1α-Gsα^R201C^* mice (Figure 3A). In this model, which developed a FD skeletal phenotype completely overlapping that of *EF1α-Gsα^R201C^* mice,^19^ all nerve fibers in the body could be traced based on the expression of GFP. Indeed, preliminary analysis of peripheral organs such as eyes, skin, gut and skeletal muscle confirmed the presence of GFP-expressing nerve fibers. Interestingly, we did not observe any change in the innervation of these organs between *BAF53b-GFP* and *BAF53b-GFP;EF1α-Gsα^R201C^* mice (Figure S3A).

**Figure 4:**
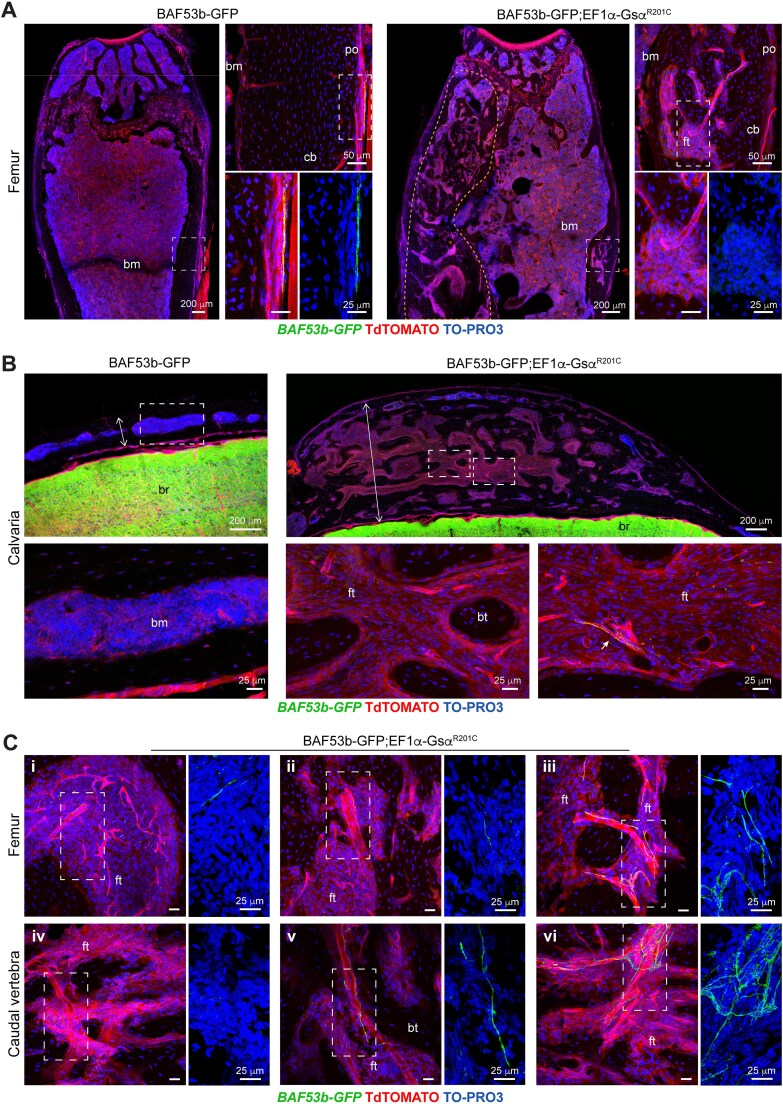
Localization of GFP-positive nerve fibers in mouse FD lesions. (A) Low and high magnification images of femurs, showing the lack of an evident innervation in these FD lesions of *BAF53b-GFP;EF1α-Gsα^R201C^* mice. FD lesion in the femur of *BAF53b-GFP;EF1α-Gsα^R201C^* mouse is delimited by the orange dotted line. (B) Low and high magnification images of calvariae, showing a drastic morphological change of the bone microarchitecture due to the development of FD in *BAF53b-GFP;EF1α-Gsα^R201C^* mice, and the presence of rare nerve fibers within the fibrous tissue (arrow). Note the markedly increased thickness of the calvarial bone in *BAF53b-GFP;EF1α-Gsα^R201C^* mouse. (C) Representative high magnification images taken in the metaphyseal regions of femurs (i-iii) and throughout the caudal vertebra (iv-vi) showing the high heterogeneity in the presence of GFP-positive nerve fibers in the fibrotic marrow of *BAF53b-GFP;EF1α-Gsα^R201C^* mice. Please note the intimate and necessary association with large caliber blood vessels. Z-stacks of the confocal images are 60 μm thick. Abbreviations: po, periosteum; cb, cortical bone; bm, bone marrow; ft, fibrous tissue; bt, bone trabecula; br, brain.

In femurs and caudal vertebrae of *BAF53-GFP* control mice, nerve fibers were found mainly in the fibrous layer of the periosteum (Figures 3B and 4A). GFP-positive fibers were also detected in the hematopoietic bone marrow of femurs and in the fatty bone marrow of caudal vertebrae (Figure 3B). In contrast, they were only rarely found in the cortical bone of the same skeletal segments (Figure S3B). As expected, nerve fibers were never observed in the growth plate (Figure S3C). In *BAF53b-GFP;EF1α-Gsα^R201C^* mice, the pattern of bone innervation in skeletal compartment not involved by FD, was overall similar to that of control mice, with periosteum being the most innervated compartment (Figure 3B). We never observed sprouting of nerve fibers.

**Figure 5:**
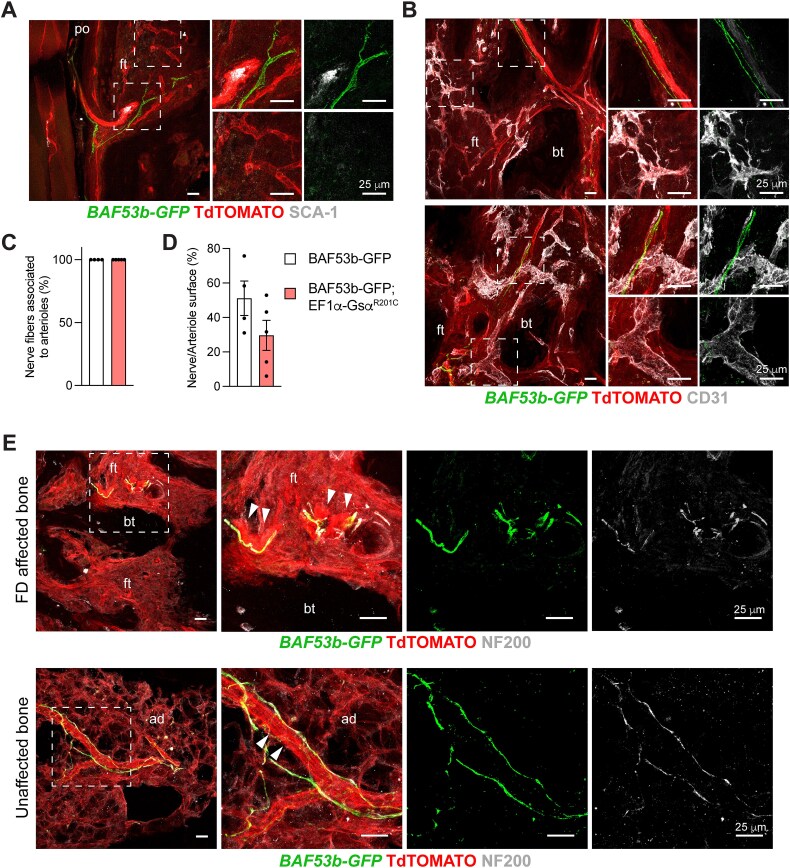
Association of nerve fibers with arterial blood vessels in mouse FD lesions. (A, B) Representative confocal images of FD lesions from caudal vertebrae of 8-mo-old *BAF53b-GFP;EF1α-Gsα^R201C^* mice immunostained with SCA-1 (A) and CD31 (B) antibodies. GFP-positive nerve fibers are predominantly associated with SCA-1-positive arteries/arterioles within the fibrotic marrow. (C) Quantification of nerve fibers associated with arteriolar surface. Please note that all the nerve fibers in *BAF53b-GFP* and *BAF53b-GFP;EF1α-Gsα^R201C^* mice were found next to arterial blood vessels. (D) Percentage of nerve fiber surface on arteriolar surface. Note the lower ratio in *BAF53b-GFP;EF1α-Gsα^R201C^* mice compared to *BAF53b-GFP* control mice, indicating that rich vascularization in FD lesions is not necessarily accompanied by nerve fiber growth. (E) NF200 immunolocalization in affected and unaffected caudal vertebrae from 8-mo-old *BAF53b-GFP;EF1α-Gsα^R201C^* mice. The colocalization of GFP-positive nerve fibers with NF200 demonstrates their sensory nature. No overall differences in nerve distribution are observed between vertebrae with FD lesions and those with normal bone marrow containing adipocytes. Abbreviations: po, periosteum; ft, fibrous tissue; bt, bone trabecula; ad, bone marrow adipose tissue.

The distribution of nerve fibers within the FD tissue was analyzed in lesions of different skeletal segments, such as femurs, calvariae, jaws, and caudal vertebrae. Overall, we did not observe a massive innervation of FD lesions (Figures 4A-C and S3D). Indeed, GFP-positive nerve fibers were focally detected in the fibrotic marrow (Figure 4A-C), showing mainly a linear morphology (Figure 4B and C) and a regular distribution along the blood vessel wall (Figure 4B, C and 5A). According, images reminiscent of nerve fiber sprouting were observed rarely and only at sites of blood vessel branching (Figure 4Ciii and vi). We never observed neuroma-like structures.

SCA-1 and CD31 immunolocalization showed that GFP-expressing nerve fibers were restricted to the wall of arterial blood vessels (Figure 5A and B). Quantification of nerve and blood vessel profiles further supported the indispensable perivascular distribution of nerve fibers in the fibrous tissue since all GFP-positive axons were found to be associated to arteries/arterioles (Figure 5C). Interestingly, the fraction of the vessel wall surface coated by nerve fibers was lower in FD lesions of *BAF53b-GFP;EF1α-Gsα^R201C^* mice compared to bone marrow of control mice (Figure 5D) indicating that the florid vascularization of the FD tissue is not necessarily associated with the expansion of the peripheral nerve network.

### Sensory nerve fibers were found within mouse FD bone lesions

Since GFP labeling in *BAF53b-GFP;EF1α-Gsα^R201C^* mice did not allow to distinguish the different types of nerve fibers, the presence of sensory innervation was assessed by immunolocalization studies based on the expression of NF200, CGRP and TRKA.

NF200 expression was found in FD lesions and unaffected skeletal segments of *BAF53b-GFP;EF1α-Gsα^R201C^* mice and colocalized with the GFP-positive nerve fibers associated with arterial vessels (Figure 5E).

**Figure 6:**
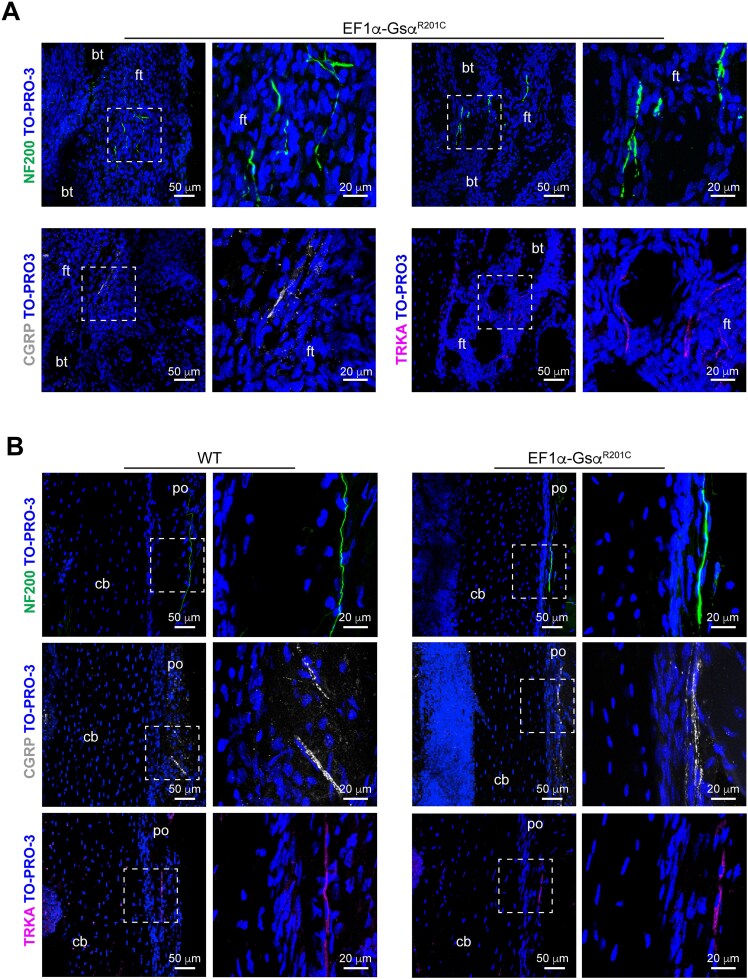
Sensory nerve fibers in mouse FD lesions. (A) Representative confocal images of tibial FD lesions of *EF1α-Gsα^R201C^* mice showing NF200, CGRP and TRKA-positive nerve fibers within the pathological fibrous tissue. (B) Confocal images of tibial periosteum of WT littermates and *EF1α-Gsα^R201C^* mice immunostained with NF200, CGRP and TRKA antibodies showing similar representation of the different nerve markers in the 2 groups of mice. Abbreviations: po, periosteum; cb, cortical bone; ft, fibrous tissue; bt, bone trabecula.

Along with NF200, CGRP and TRKA were also found to be expressed by the nerve fibers within the fibrous tissue of FD lesions (Figure 6A). Interestingly, we observed that the pattern of immunoreactivity was overall comparable in periosteum, cortical bone and bone marrow of WT and *EF1α-Gsα^R201C^* mice (Figure 6B and S4A, B). Altogether, these results demonstrated that sensory nerve fibers could be found within mouse FD lesions, but no clear signs of pathological rearrangement were found either in the FD lesions or in unaffected compartments.

### Human FD bone lesions did not show pathological sprouting of sensory nerve fibers

**Figure 7:**
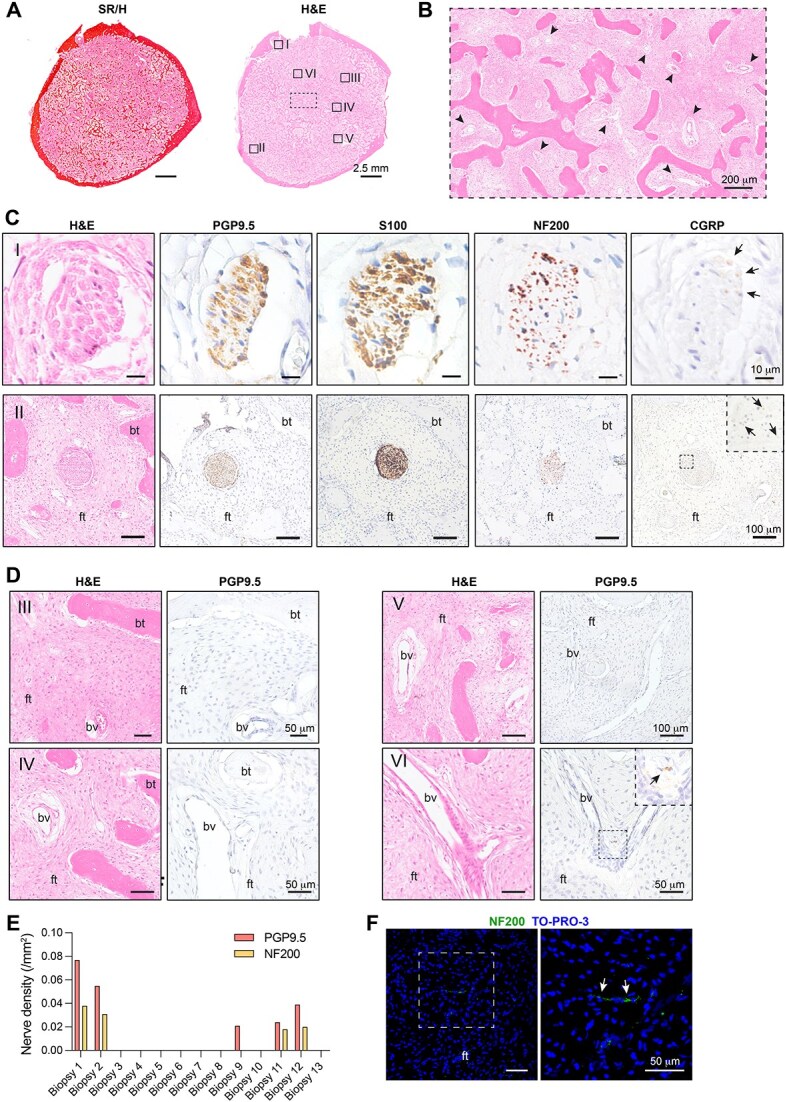
Pattern of innervation in human FD lesions. (A) Representative sirius red/hematoxylin (SR/H) and H&E-stained transverse section of a human tibia with FD. The dotted box is a reference for the image in B. The boxes (I-VI) are references for the images in C and D. (B) High magnification image of the H&E-stained section, showing the rich vascularization (arrowheads) of the FD fibrous tissue. (C) Images from 3-μm-thick paraffin sections stained with H&E and immunostained with PGP9.5, S100, NF200, and CGRP showing intralesional peripheral nerve bundles identified at the endosteal area of the sample (I and II). While NF200 was widely expressed in the fibers within the nerve trunks, CGRP labeling was found in a lower number of axons (arrows). (D) Representative images from 3-μm-thick paraffin sections stained with H&E or immunostained with PGP9.5 showing large areas of fibrous tissue without (III-V) or with rare (VI, arrow) nerve fibers associated to blood vessels. (E) Quantification of the PGP9.5 and NF200-positive nerve density found in the 13 FD bone biopsies. The count of nerve bundles was normalized on the biopsy tissue area. (F) Representative images from 60-μm-thick gelatin sections immunostained with NF200, showing very rare, thin nerve fibers in the fibrous tissue (arrows). Abbreviations: ft, fibrous tissue; bt, bone trabecula; bv, blood vessels.

We then investigated the innervation pattern of 13 bone biopsies obtained from different skeletal sites of 7 patients diagnosed with FD/MAS. The biopsy area ranged from 9.73 to 291.12 mm^2^ with the largest representing a whole-mount transverse section of a tibia (Figure 7A). All samples showed the typical highly vascularized fibro-osseous tissue replacing normal bone and marrow (Figure 7A and B). Immunolocalization of the pan-neuronal marker PGP9.5 on paraffin-embedded sections of a HD and a non-lesional bone sample from a FD patient showed specific nerve fiber staining in all the different skeletal compartments (Figure S5A and B).

In FD samples, PGP9.5 immunoreactivity was detected in intralesional nerve trunks associated with large blood vessels near the cortical bone, which also reacted with S100 highlighting myelinated axons (Figure 7C). Within the same structures, sensory markers as NF200 and CGRP were also expressed, although the latter stained a lower number of axons (Figure 7C).

Within the fibrotic marrow, we observed large areas devoid of nerve fibers. Indeed, despite the rich network of blood vessels detected in all lesions, only few PGP9.5- and NF200-expressing nerve bundles were found (Figure 7D and E, S6A and B). These structures were discrete, well defined and did not show evidence of unorganized axon fiber growth (Figure 7C and S6A, B).

Immunolocalization of NF200 was also performed on thick gelatin-embedded sections followed by confocal Z-stack analysis, which revealed within the fibrotic marrow only rare and thin nerve fibers (Figure 7F). To complete the analysis of the different types of nerve fibers, we stained sections with an anti-TH antibody and showed that few sympathetic nerves could be detected either within nerve fascicles or around the blood vessel wall (Figure S7A). These experiments demonstrate that in FD lesions, at least from young patients, aberrant nerve sprouting or formation of neuroma-like structures is not a pathological feature of the disease.

Since we previously observed that the levels of *BDNF*, a neurotrophin involved in pain mechanisms, is increased in FD tissue compared to normal bone, we expanded the analysis to other neurotrophins (Figure S7B). Confirming our previous results, *BDNF* transcripts were higher in FD than in HD bone samples, although the result did not reach the statistical significance due to the high variability.^17^ Similarly, *NGF* expression was higher in FD compared to HD, although variability was observed among the different FD samples. No differences were observed in *NTF3* expression, while *NTF4* expression was undetectable in both FD and HD bone samples (Figure S7B).

## Discussion

Pain is defined as “an unpleasant sensory and emotional experience associated with, or resembling that associated with, actual or potential tissue damage” (https://www.iasp-pain.org/). Chronic bone pain is a clinical feature of many genetic skeletal diseases but in most of them the driving mechanisms remain unknown. This gap of knowledge has multiple reasons, including the technical hurdle in studying bone and bone marrow innervation and the difficulty in gathering large cohorts of patients with rare bone diseases.

FD is a genetic disorder in which bone pain is often caused by bone deformity and fracture, as in appendicular bones^30^^,^^31^ or nerve impingement, as in craniofacial bones.^32^ However, even in the absence of these complications, many FD patients complain of an “intrinsic” chronic pain,^8^^,^^9^^,^^11^^,^^33^^,^^34^ which in a subset of patients is of neuropathic type.^9^ Interestingly, the skeletal disease burden, which correlates with the patient’s quality of life and illness perception,^33^ is predictive for the development of pain^34^ but does not affect the pain score.^8^^,^^9^^,^^35^ Therefore, understanding the physio-pathological bases of the unpleasant sensory experience associated with this disease is particularly intricate.

The *EF1α-Gsα^R201C^* mouse is currently the animal model that more closely reproduces the natural history of the human bone disease.^19^ In FD patients, bone lesions are not detected at birth^36^ and appear at variable times and sites during skeletal growth.^32^ Similarly, in *EF1α-Gsα^R201C^* mice bone lesions develop spontaneously and asynchronously in the post-natal life and generate different patterns of skeletal involvement. Therefore, these mice offer a suitable model for correlation studies between disease burden and other clinical or biochemical parameters. Furthermore, due to the ubiquitous expression of the transgene, they allow to investigate the cell-autonomous effects of the Gsα mutation on any cell type and compartment, including the peripheral and central nervous systems.

In this study, we first analyzed *EF1α-Gsα^R201C^* mouse well-being using the burrowing and nesting tests which are also commonly used to assess the spontaneous pain-like behavior.^22^

We observed a clear difference between *EF1α-Gsα^R201C^* and WT mice, with the former showing a reduced burrowing and nesting capacity as soon as 3 mo of age. This behavior did not depend on the number and distribution of FD lesions and, at least in the burrowing test, appeared earlier in female compared to male mice. As shown in the correlation analyses between disease burden scores and behavioral tests, mice with low disease burden, which is usually associated with lesions restricted to caudal vertebrae, could experience highly impaired behavioral functions. On the contrary, mice with high disease burden could behave like WT mice. This is consistent with the notion that the severity of the skeletal phenotype is not the main pain-determinant factor in FD.^9^^,^^37^ Furthermore, no correlation was overall observed among the different tests indicating that they were able to reflect different behavioral patterns. These results suggest that the behavioral impairment was not due to a hinderance of movements caused by FD lesions in bones, neither to a general malaise of the mice. However, due to the ubiquitous expression of the transgene in the *EF1α-Gsα^R201C^* model, we cannot exclude an effect of the Gsα mutation on muscle performance. Of note, burrowing activity and nest construction in mice are comparable to human functions of daily living including care of the self and interaction with domestic and social environment.^23^ Therefore, the impaired burrowing and nesting capacity of *EF1α-Gsα^R201C^* mice seems to reflect the overall well-being impairment that may be caused by FD in human patients.^38^ Similarly, the scores of the tail flick test, which measures the pain threshold in rodents,^26^ were in complete agreement with data from human FD patients. Indeed, a recent work by Golden et al. reported a higher pain tolerance to heat and cold stimuli in FD/MAS patients compared to controls.^10^ Of note, the authors of this study properly raised the point of the origin of tolerance to noxious stimuli in FD patients, asking whether it might result from the continuous use of analgesics or from an adaptation process to persistent pain. The analysis of our transgenic mice, which did not receive any analgesic treatment, seems to support the latter possibility.

We then investigated, for the first time, the pattern of bone innervation in FD. To this aim, on the one side we developed a new *EF1α-Gsα^R201C^* mouse model expressing a reporter gene under the control of a pan-neuronal promoter; on the other side, we analyzed the innervation status of a series of human FD lesions. Lineage tracing studies clearly demonstrated that the overall amount and distribution of nerve fibers in organs and bones of mice with mutated Gsα were comparable to those of controls. Within mouse FD bone lesions, sensory innervation consisted essentially in linear nerve fibers that were associated with arterial blood vessels.

In human FD biopsies, despite the rich vascularization of the fibrotic tissue, a low number of intralesional nerves was detected by the pan-neuronal marker PGP9.5. Part of the nerve fibers were sensory in nature as highlighted by NF200 immunostaining. CGRP-positive fibers were also found, although staining was weaker and restricted to few axons within the large nerve bundles. Furthermore, no abnormalities were observed in the distribution of sympathetic nerve fibers.

Although this study does not clarify whether the intralesional nerves were normal preexisting structures entrapped within the lesional tissue, altogether, our data seem to dispel the hypothesis that FD stimulates irregular sprouting of sensory fibers as observed in bone cancer,^2^^,^^39^^,^^40^ despite the local expression of neurotrophic factors.

The scarce representation of intralesional afferent nerve fibers transmitting signals to CNS seems to be consistent with the lack of correlation between pain and disease burden that characterizes the disease. Undoubtedly, however, additional mechanisms are required to generate the unpleasant sensation experienced by FD patients. Previous work showed that the anatomical distribution, rather than the number and size of FD lesions, is an important determinant in the clinical expression of the disease. Indeed, painful lesions are more frequently observed in lower extremities and ribs compared to upper extremities and craniofacial bones.^34^ The relevance of lesion topography could depend on the size or density of pre-existing nerves in affected skeletal segments^1^ as in thoracic lesions, and/or the presence of mechanical stress, as in lesions of lower extremities.^34^ However, it must be noted that very different pain scores were reported in patients with craniofacial FD and comparable images of trigeminal compression and displacement.^35^ Similarly, we did not observe a clear connection between mouse behavioral scores and the anatomical distribution of FD. Specific pathological changes and/or inflammatory cytokine secretion within individual FD lesions may also be implicated in the generation of an algogenic microenvironment. Inflammatory cytokines such as IL-6 have been shown to be produced by the fibrous tissue.^41^ However, IL-6 is not elevated in the circulation,^42^ and neither IL-6 inhibition nor other anti-inflammatory drugs, decrease significantly bone pain in FD patients.^11^^,^^43^ Importantly, inflammatory cells are never found within the FD lesion microenvironment, except for mastocytes.^44^ Enhanced bone resorption, which is a recurrent feature of FD, may also play a role by generating an acidic environment that stimulates sensory neurons.^45^ Since the number of osteoclasts varies among different FD lesions,^17^^,^^29^ it is likely that lesions with active bone resorption result in higher levels of acidity and enhanced nerve stimulation compared to quiescent ones. However, pain-relief is not achieved in all FD patients treated with anti-bone resorption drugs.^12–14^^,^^46^^,^^47^ Furthermore, osteomalacia may contribute to the pain sensation in FD, as inhibition of FGF23 using burosumab was accompanied by pain reduction in a child and an adult patient with severe FD/MAS.^48^^,^^49^ Finally, considering the somatic condition of the human disease, the presence of unpleasant sensation reported by some patients might depend on the expression of the Gsα mutation in critical components of the nervous system. Our analyses of FD mouse skeleton and organs seem to exclude a cell-autonomous effect of the mutation on the growth and structure of peripheral nerves. However, it remains to assess whether the mutation alters the function of sensory nerves and/or it affects the organization and activity of the CNS, where pain-related information is elaborated. In this context future studies investigating the expression of molecular pain mediators in CNS are needed. Interestingly, Golden et al. recently analyzed a cohort of FD patients using diffusor tension imaging and observed a structural compromission of some withe matter pathways that are critical to personality and other feelings such as depression and anxiety.^10^ Although discordant reports are found in literature about the role of these feelings in FD, the work by Golden and colleagues suggests that the sensation of chronic pain in FD patients may result from the complex integration of physical, psychological and neurobiological mechanisms. This would be consistent with a nociplastic type of pain, in which altered nociception is not explained by tissue damage or pathological alteration of the sensory system (https://www.iasp-pain.org/).^50^ However, the role played by psychological states and negative feelings caused by self or societal stigma is difficult to assess in mice.

In conclusion, we showed that the *EF1α-Gsα^R201C^* mouse model of human FD is characterized by evident behavioral deficits. Furthermore, we demonstrated for the first time that, albeit sensory fibers are found within FD lesions and may contribute to bone pain, abnormal nerve sprouting and formation of neuroma-like structures are not pathological features of this disease. This is a preliminary study that has evident limitations, including the absence of data on FD mouse CNS, the low number of human FD biopsies and the absence of rigorous scale-based evaluation of pain in the FD patients from which they were obtained. Nonetheless, it provides novel findings that will help to better characterize the unpleasant sensory experience associated with FD.

## Supplementary Material

4)_Supplementary_information_R1_clean_version_zjaf066

## Data Availability

Data will be available from the corresponding author upon request.
