# Supplementary material for: Bone pain in fibrous dysplasia does not rely on aberrant sensory nerve sprouting or neuroma formation
Source: J Bone Miner Res. 2025 May 5;40(8):999–1014. doi: 10.1093/jbmr/zjaf066 (PMC12308826; doi:10.1093/jbmr/zjaf066)
Supplement: 4)_Supplementary_information_R1_clean_version_zjaf066 [file 4)_supplementary_information_r1_clean_version_zjaf066.docx]

**Supplementary data**

**Figure S1**

**
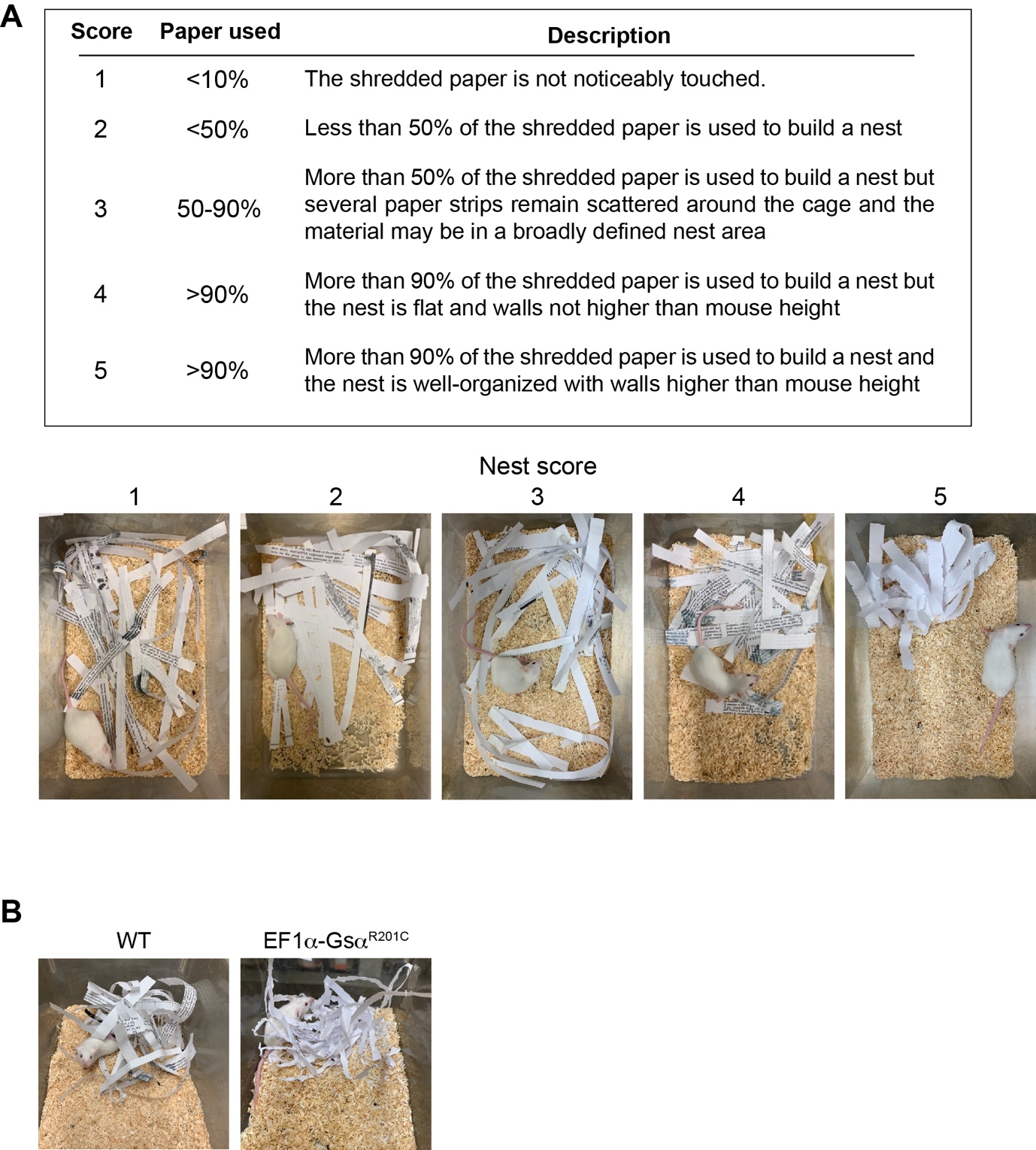
**

**Figure S1. Nesting test scoring system**. A) Table with scoring and example pictures of the nests. B) Representative high scored nests from WT and EF1α-Gsα^R201C^ mice. Note that the shredded paper used for the nest by the EF1α-Gsα^R201C^ mouse resulted chewed.**Figure S2**

**
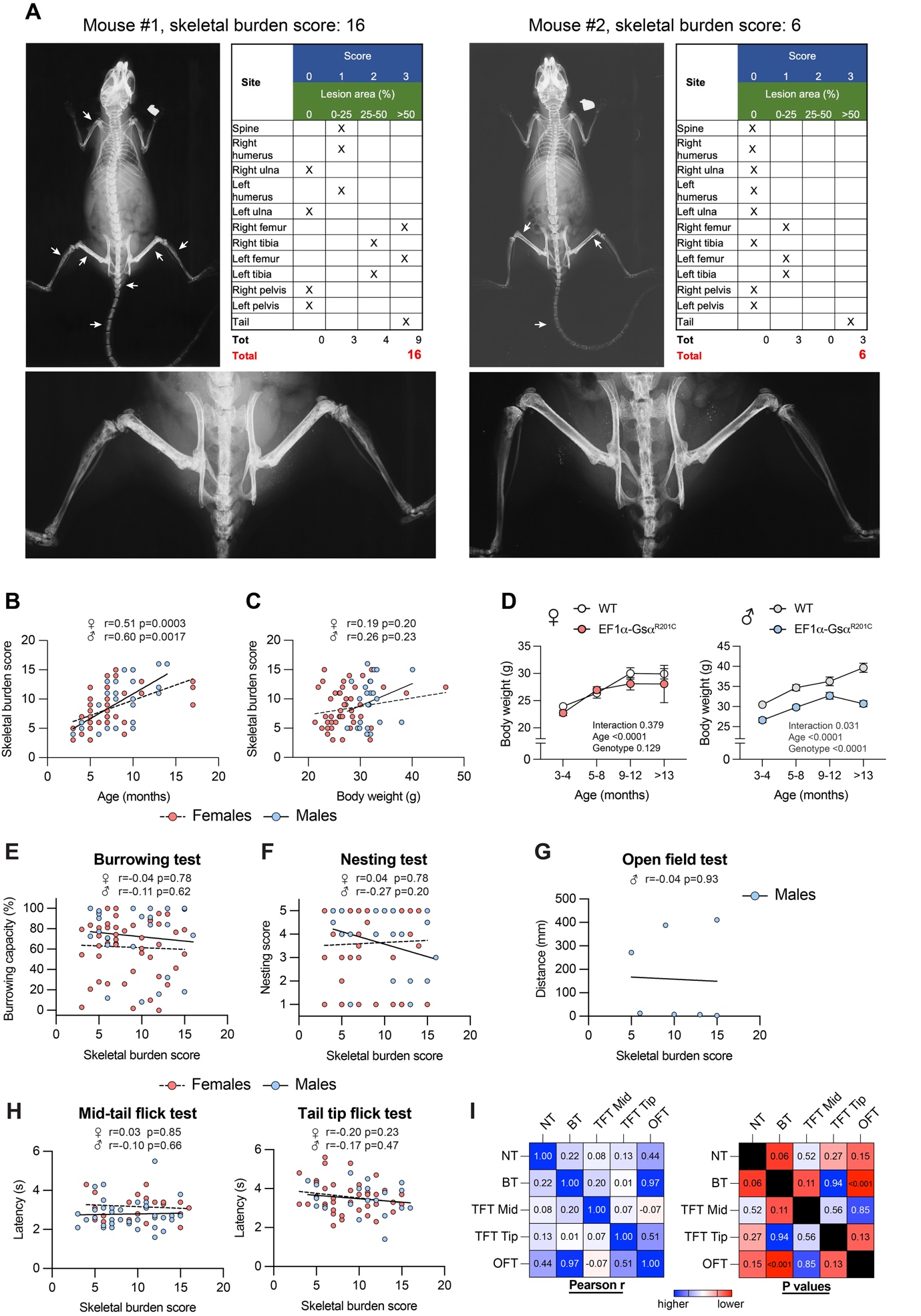
**

**Figure S2. Skeletal burden score evaluation and correlation with pain behavior and other parameters in EF1α-Gsα^R201C^ mice**. A) Radiograms and table for evaluation of the skeletal disease burden. In these pictures, two representative mice are shown with different scores. Arrows indicate FD lesions. B) Correlation analysis between burden scores and age. C) Correlation analysis between burden scores and body weight. D) Measurements of body weight in female and male WT and EF1α-Gsα^R201C^ mice at different ages. P-values from the two-way ANOVA analysis are reported in each graph. E) Correlation analysis between burrowing capacity and skeletal burden score. F) Correlation analysis between nesting capacity and burden scores. G) Correlation analysis between open field test results and burden scores. H) Correlation analysis between tail flick latency times and burden scores.

In B, C and E-H correlation coefficient (r) and p-value are shown above each graph. Please note that mice with very low disease burden scores can show poor burrowing, nesting and locomotor ability; similarly, mice with very high disease burden scores may not experience any pain-like behavior. I) Correlation matrix analysis among the different behavioral tests. Pearson r coefficient and p-values are shown in two different graphs. Note that no strong relationships are observed among the tests, except between burrowing and open field tests, for which a positive correlation is detected. NT=Nesting test, BT=burrowing test, TFT Mid=tail flick test mid-tail, TFT Tip=tail flick test tail tip, OFT=open field test.

**Figure S3**


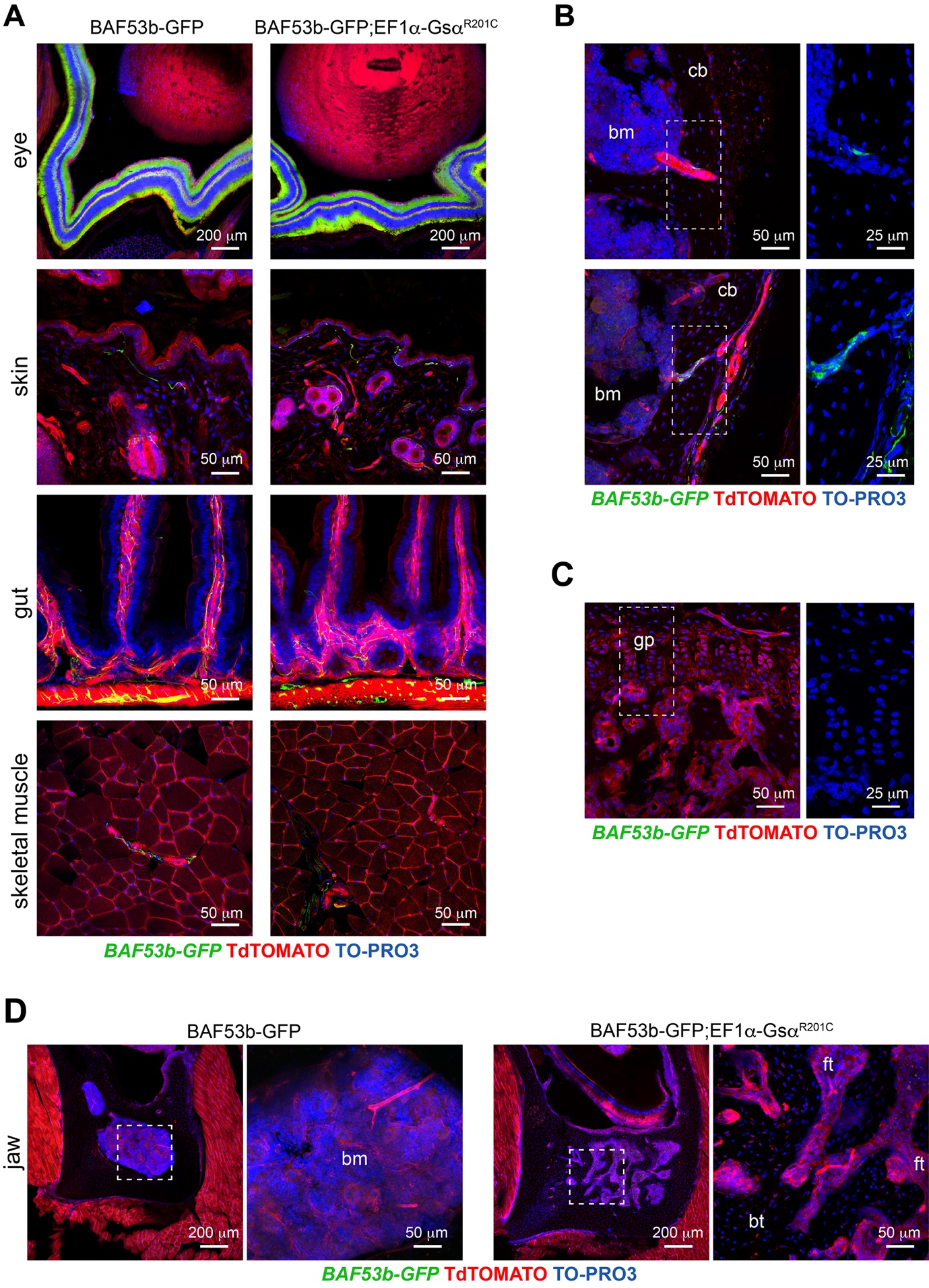


**Figure S3. Pattern of Baf53b-GFP tracing in different organs and skeletal compartments.** A) GFP labeling in some peripheral organs, revealing no overall differences between *BAF53b*-*GFP* and *BAF53b-GFP;EF1α-Gsα^R201C^* mice. B) Representative pictures showing GFP+ intracortical nerve fibers in *BAF53b*-*GFP* mice. C) Representative pictures showing the absence of nerve fibers in the cartilaginous growth plate and trabecular bone. D) Pictures of jaw bones showing no nerve fibers neither in the hematopoietic bone marrow of *BAF53b*-*GFP* mice nor in fibro-osseous lesions fo *BAF53b-GFP;EF1α-Gsα^R201C^* mice. Except for the muscle images showing a single plane, Z-stacks of the confocal images are 60 μm thick. cb=cortical bone, bm=bone marrow, ft=fibrous tissue, bt=bone trabecula, gp=growth plate.

**Figure S4**

**
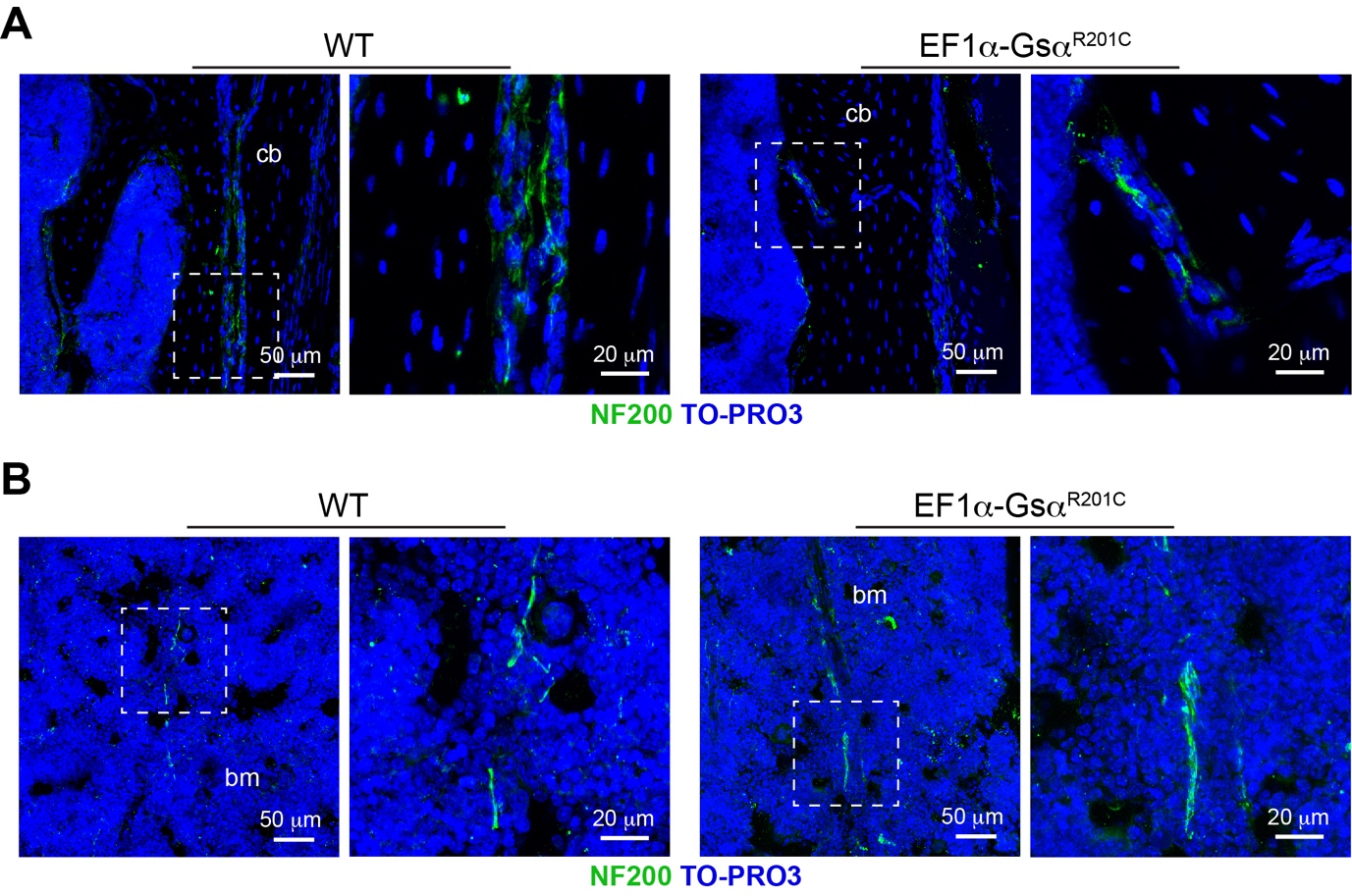
**

**Figure S4. Similar pattern of sensory innervation in cortical bone and bone marrow in WT and EF1α-Gsα^R201C^ mice.** A) Representative NF200 immunostaining of tibial cortical bone. B) Representative NF200 immunostaining of hematopoietic bone marrow showing linear nerve fibers in both genotypes. Z-stacks of the confocal images are 60 μm thick. cb=cortical bone, bm=bone marrow.

**Figure S5.**

**
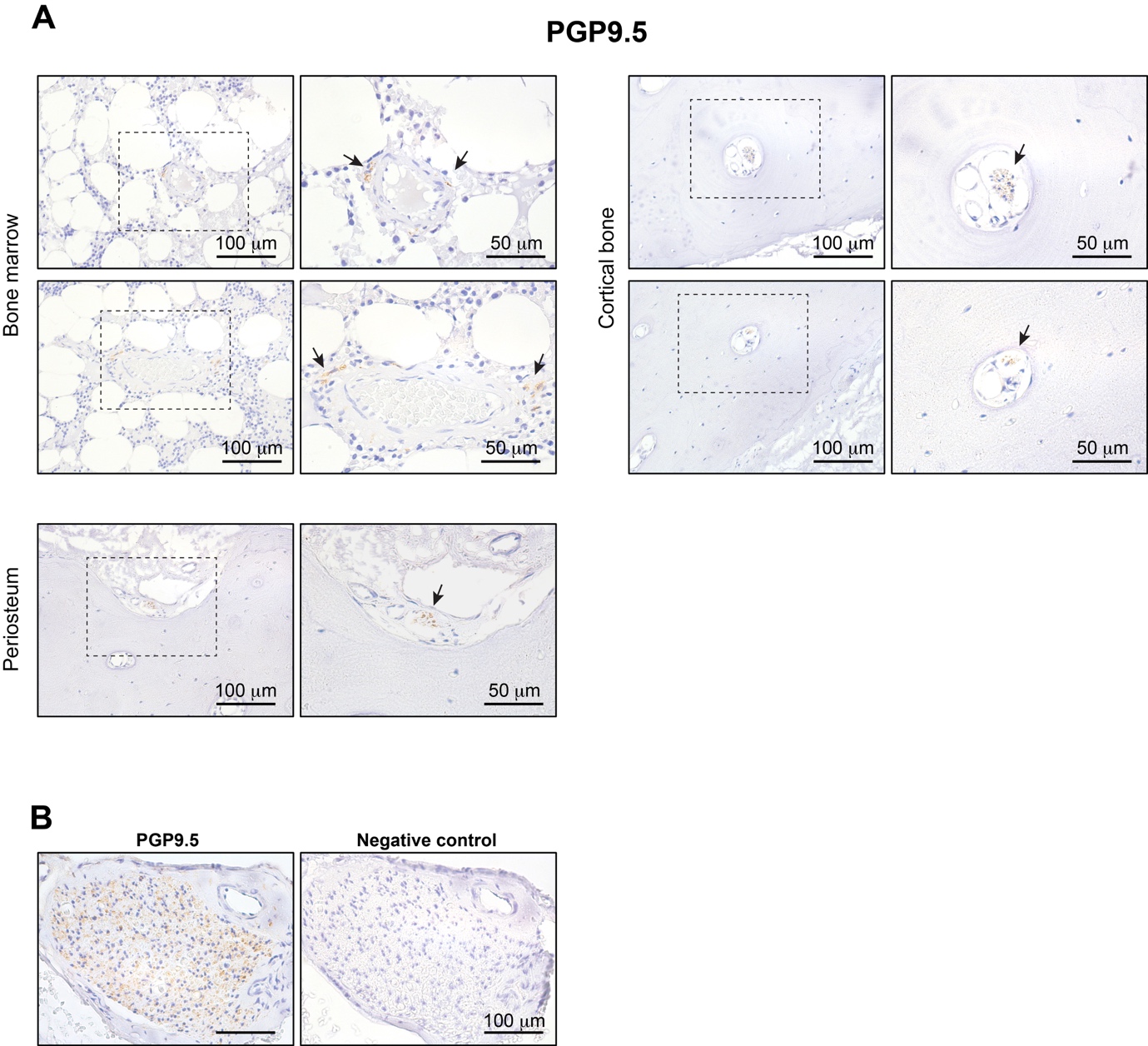
**

**Figure S5.** A) Representative images from samples the HD tibia (bone marrow) and FD unaffected fibula (cortical bone and periosteum) showing PGP9.5 expression in nerve fibers. B) PGP9.5 immunohistochemistry of a nerve trunk with the negative control lacking the primary antibody.

**Figure S6.**

**
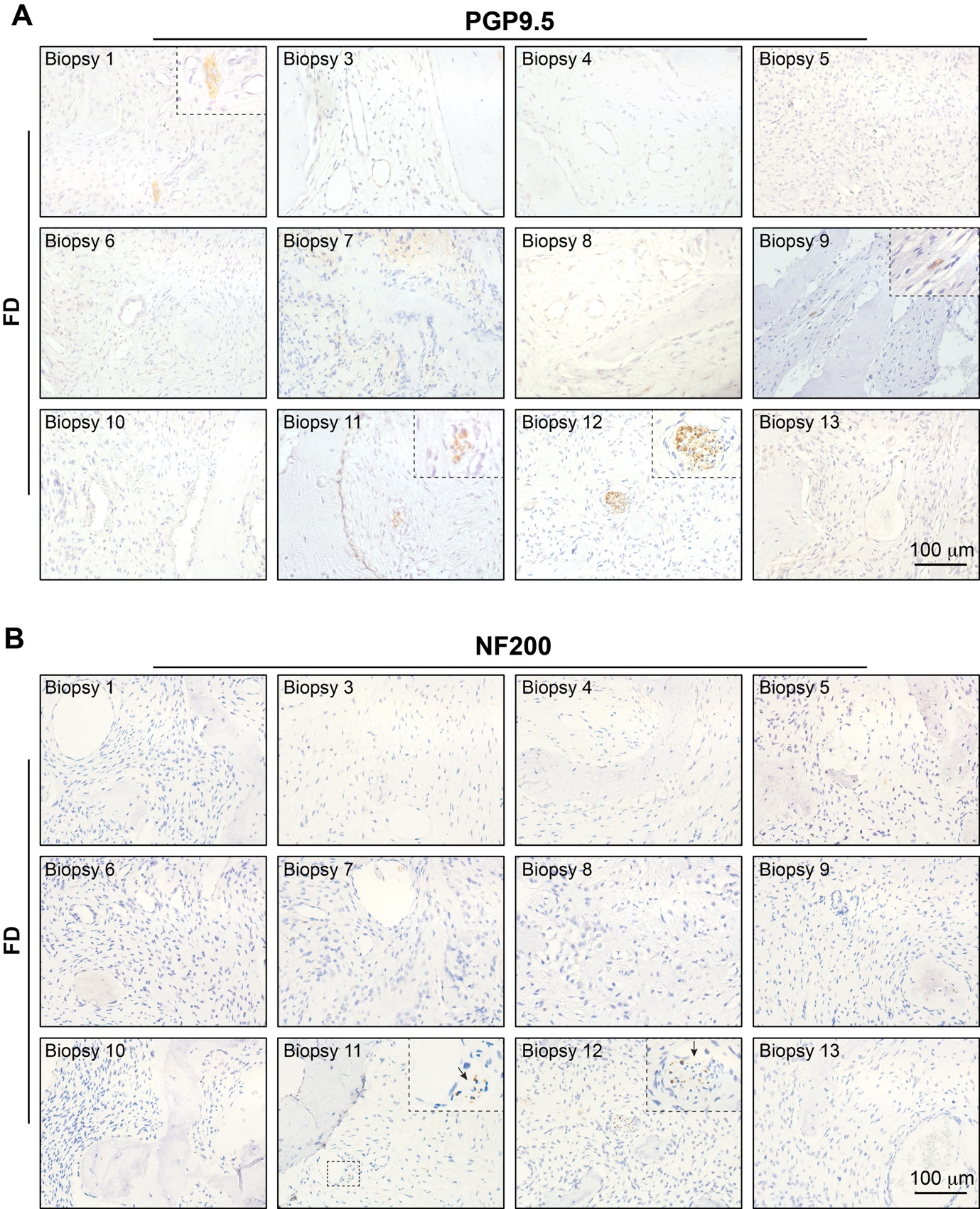
**

**Figure S6.** A) Representative images of all the 13 human FD bone biopsies immunostained with PGP9.5 antibody and B) with NF200 antibody showing few nerves within the FD lesions. Please note that Biopsy 2 is shown in Fig. 7.

**Figure S7.**

**
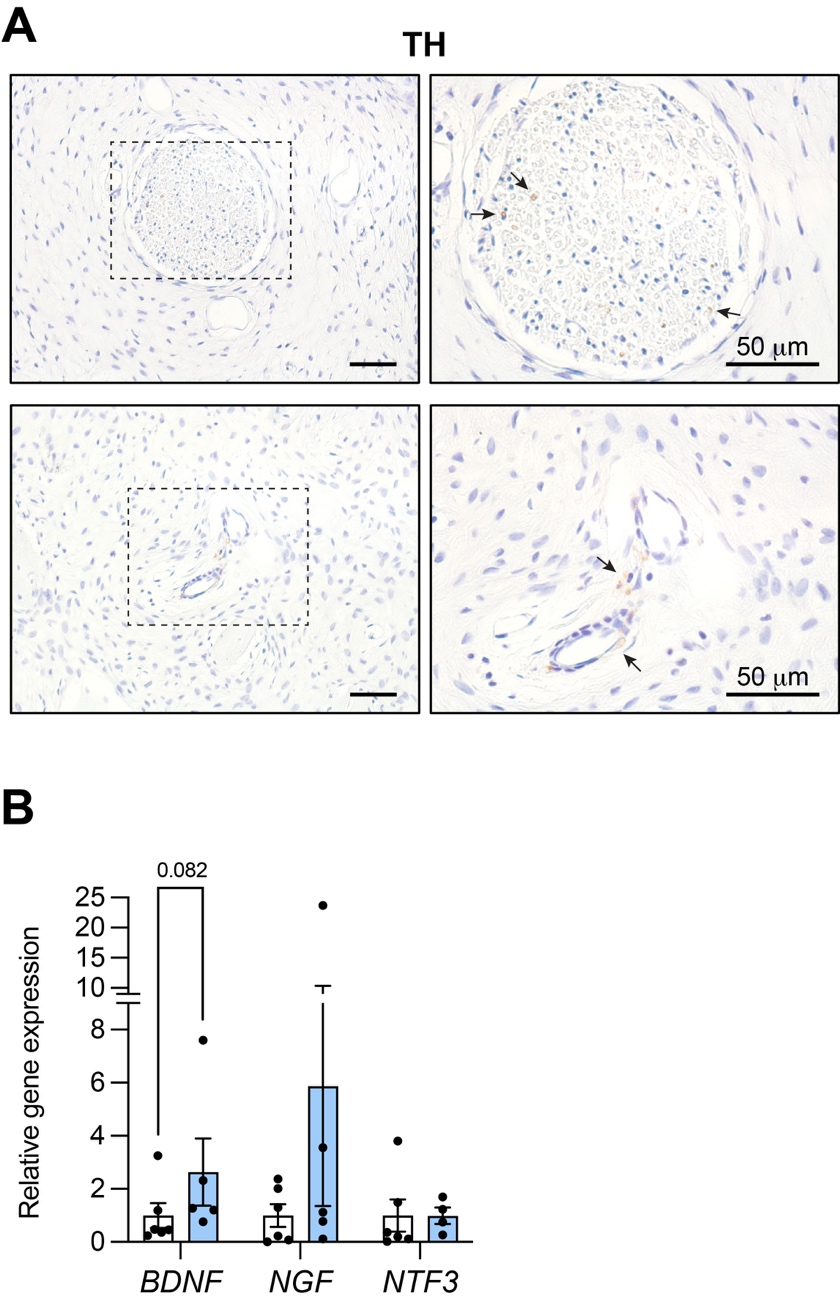
**

**Figure S7.** A) Representative images of nerves within FD lesions immunostained with TH antibody, showing the staining of sympathetic fibers within a nerve bundle or in close association to a blood vessel (arrows). B) qPCR gene expression analysis of neurotrophins performed on fresh healthy donors (HD) and FD bone tissues. *RNA18SN5* was used as housekeeping gene for normalization. Data are shown as dot plots with column bars showing all the experimental samples. Statistical analysis was performed using Student t-test and exact p-value for *BDNF* panels is reported.**Table S1**
